# A collaborative, computer-assisted, psychoeducational intervention for depressed patients with chronic disease at primary care: protocol for a cluster-randomized controlled trial

**DOI:** 10.1192/j.eurpsy.2022.749

**Published:** 2022-09-01

**Authors:** G. Rojas, P.A. Martinez Diaz, V. Guajardo, S. Campos, P. Herrera, P. Vöhringer, V. Gomez, W. Szabo, R. Araya

**Affiliations:** 1 Hospital Clínico Universidad de Chile, Departamento De Psiquiatría Y Salud Mental, Santiago, Chile; 2 Université de Sherbrooke, Faculté De Médecine Et Des Sciences De La Santé, Longueuil, Canada; 3 Centre de recherche Charles-Le Moyne sur les innovations en santé, -, Longueuil, Canada; 4 Pontificia Universidad Católica de Chile, Nursing School, Santiago, Chile; 5 Universidad de Chile, Facultad De Ciencias Sociales, Santiago, Chile; 6 King’s College London, Centre For Global Mental Health, London, United Kingdom

**Keywords:** Chronic Diseases, e-mental health, Depression, Primary health care

## Abstract

**Introduction:**

Depression treatment recommendations seldom include chronic illness comorbidity.

**Objectives:**

To describe the rationale and methods for a cluster-randomized trial (CRT) in primary care clinics (PCC) comparing a computer-assisted psychoeducational (CAPE) intervention to usual care (UC) for depressed patients with hypertension or diabetes.

**Methods:**

Two-arm, single-blind CRT in Santiago, Chile. Eight PCC will be randomly assigned to the intervention or UC. A total of 360 depressed individuals aged 18 or older PHQ-9 scores ≥ 15 and hypertension or diabetes will be recruited. Patients with alcohol/substance abuse; current treatment for depression, bipolar disorder, or psychosis; illiteracy; severe impairment; and residents in long-term care facilities will be excluded. Patients in the intervention will receive eight CAPE sessions by trained therapists, structured telephone calls to track progress, and usual medical care for chronic diseases. Psychologists and psychiatrists will regularly supervise therapists. To ensure continuity of care, the PCC team will meet monthly with a research team member. Patients in UC will receive standard medical and depression treatment. Three, six, and twelve months after enrollment, outcomes will be assessed. The primary outcome will be a 50% reduction in baseline PHQ-9 scores at six months. Intention-to-treat analyses will be used.

**Results:**

A previous, small-scale pilot study provided valuable insights for study design.

**Conclusions:**

This study will provide first-hand evidence on the effectiveness of a CAPE for depressed patients with chronic diseases at PCC in a Latin American country.

**Disclosure:**

No significant relationships.

